# Supplementary material for: Maintenance of type 2 glycolytic myofibers with age by Mib1-Actn3 axis
Source: Nat Commun. 2021 Feb 26;12:1294. doi: 10.1038/s41467-021-21621-6 (PMC7910585; doi:10.1038/s41467-021-21621-6)
Supplement: Supplementary file 1 — Supplementary Information [file 41467_2021_21621_MOESM1_ESM.docx]

**Supplementary Information**

**Maintenance of type 2 glycolytic myofibers with age by Mib1-Actn3 axis**

Ji-Yun Seo^1^, Jong-Seol Kang^1^, Ye Lynne Kim^1^, Young-Woo Jo^1^, Ji-Hoon Kim^1^, Sang-Hyeon Hann^1^, Jieon Park^1^, Inkuk Park^1^, Hyerim Park^1^, Kyusang Yoo^1^, Joonwoo Rhee^1^, Jung-Wee Park^2^, Yong Chan Ha^2^ and Young-Yun Kong^1^*

^1^School of Biological Sciences, Seoul National University, 08826, Seoul, Korea

^2^Department of Orthopaedic Surgery, Chung-Ang University College of Medicine, 224-1 Heukseok-dong, Dongjak-gu, Seoul 156-755, South Korea

This file includes:

Supplementary Figures (1-7)

Supplementary Tables (1-3)

**
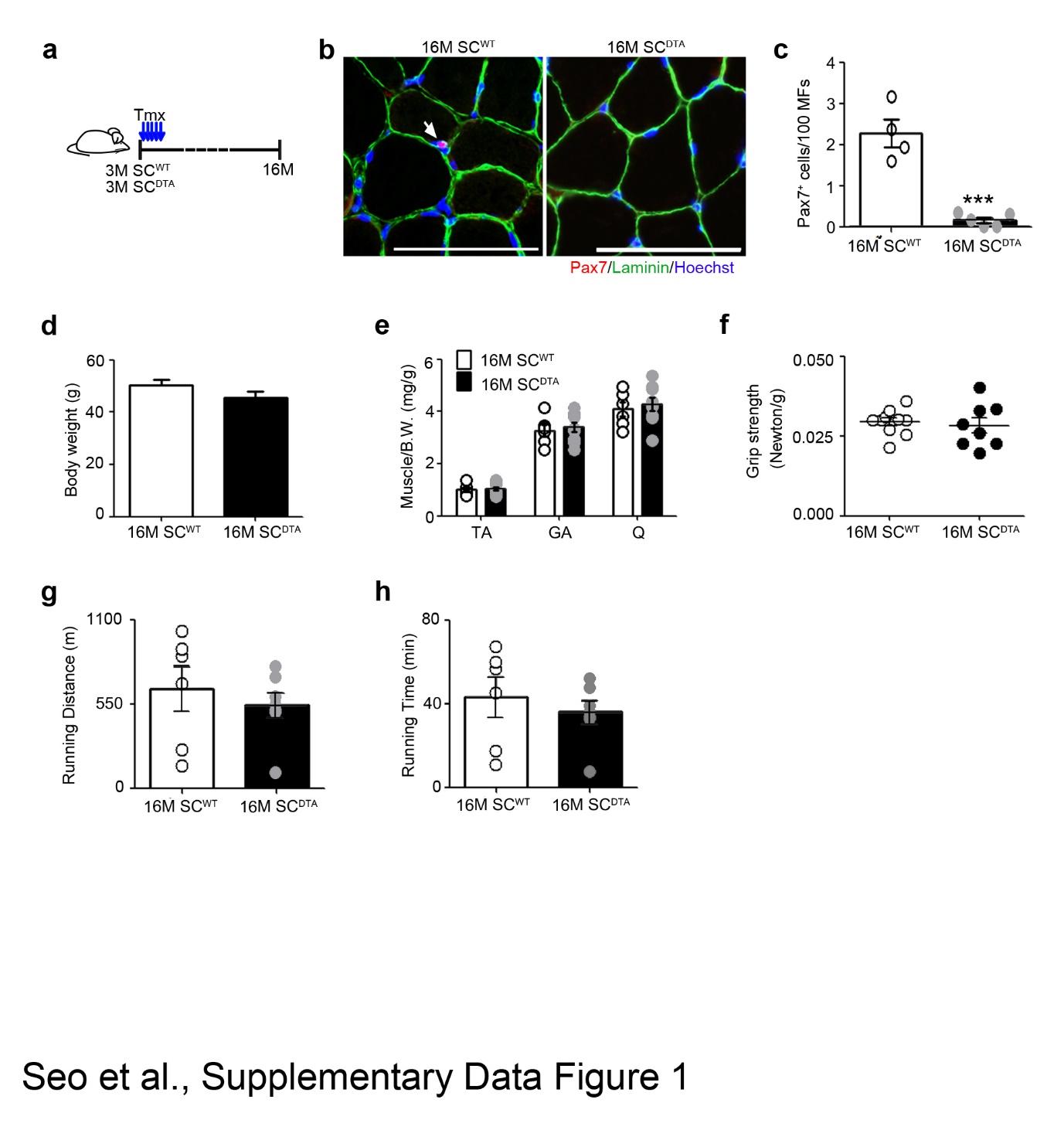
**

**Supplementary Fig 1. Dispensable role of muscle stem cells in age-associated muscle atrophy**

Three-month-old *Rosa-DTA* (SC^WT^; SC: satellite cells or muscle stem cells) and *Pax7-CreERT;Rosa-DTA* (SC^DTA^) mice were administered tamoxifen by daily oral for 5 consecutive days. TA muscles were harvested at the age of 16-month-old (**a**). **b**,**c,** Cross-sections of 16-month-old SC^WT^ and SC^DTA^ TA muscles were subjected to immunohistochemistry (IHC) staining for Pax7 (red), Laminin (green) and Hoechst (blue) (**b**), and quantification of Pax7+ cells per 100 myofibers (**c**). (*p* = 0.0003). Arrow indicates Pax7+ cells. **d,e,** Body weights (**d**) and relative hindlimb muscle mass (**e**) to body weights of 16-month-old SC^WT^ and SC^DTA^ mice. **f,** Grip strength measurement of 16-month-old SC^WT^ and SC^DTA^ mice. **g,h,** Running distance (**g**) and time (**h**) to exhaustion. Scale bars, 100 μm (**b**). Data presented are means ± s.e.m. Data shown are representatives of at least 3 independent experiments. n = 4 and 5 for SC^WT^ and SC^DTA^ mice, respectively (**c**), n = 11 and 12 for SC^WT^ and SC^DTA^ mice, respectively (**d**), n = 6 and 9 for SC^WT^ and SC^DTA^ mice, respectively (**e**), n = 10 and 8 for SC^WT^ and SC^DTA^ mice, respectively (**f**), n = 6 and 7 for SC^WT^ and SC^DTA^ mice, respectively (**g**,**h**) mice per genotype. 2-tailed Student’s *t-*test for **c**,**d**,**f**–**h**. 2-way ANOVA for **e**. ****p*<0.01.


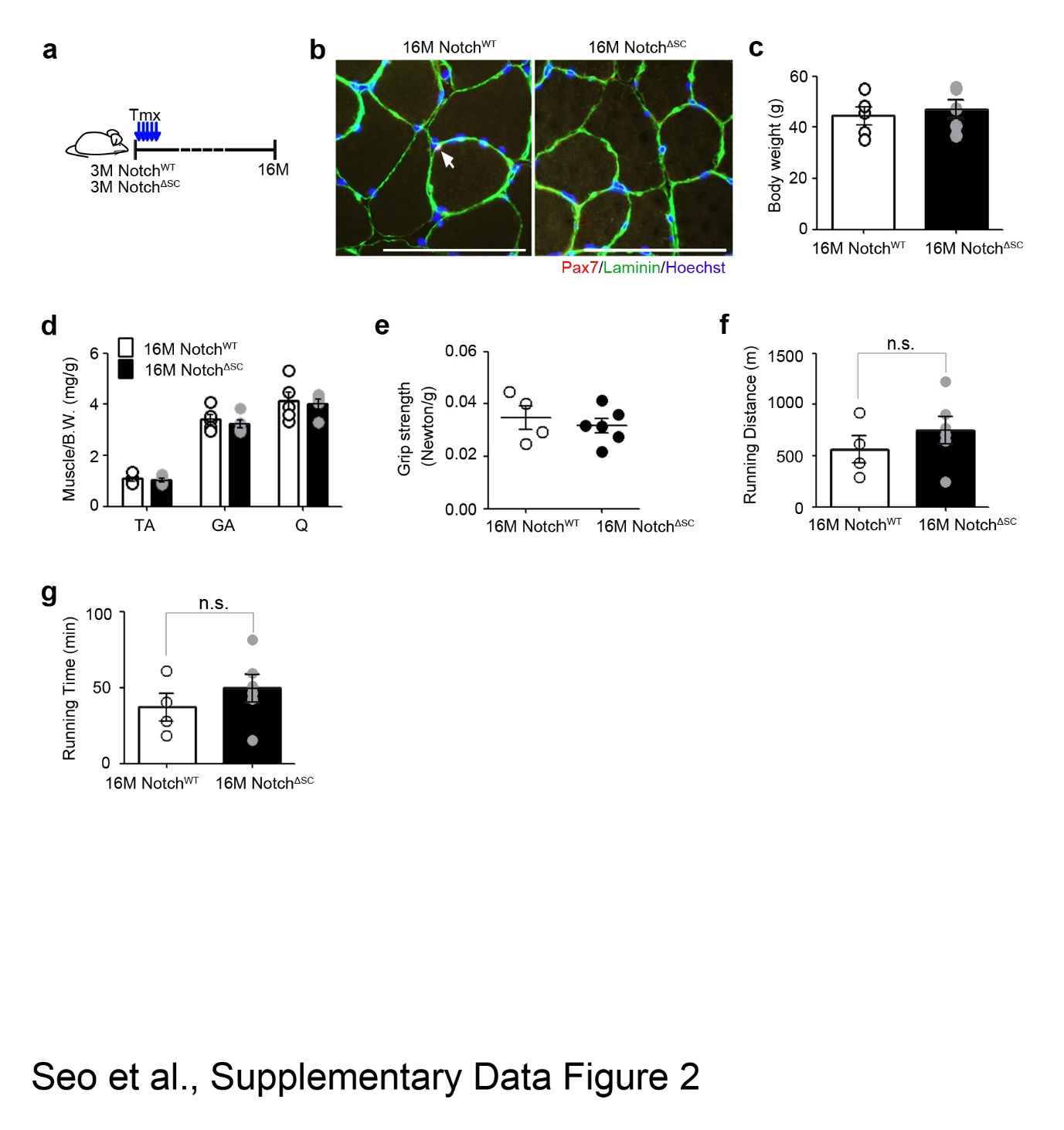


**Supplementary Fig 2. Dispensable role of Notch signaling in age-associated muscle atrophy**

Three-month old *Rbpjκ^f/f^* and *Notch1^f/f^;Notch2^f/f^* (hereafter, Notch^WT^) and *Pax7-CreERT;Rbpjκ^f/f^* and *Pax7-CreERT;Notch1^f/f^;Notch2^f/f^* (hereafter, Notch^ΔSC^) mice were administered tamoxifen by daily oral for 5 consecutive days. TA muscles were harvested at the age of 16-month-old (**a**). **b,** Cross-sections of 16-month-old Notch^WT^ and Notch^ΔSC^ TA muscles were subjected to IHC staining for Pax7 (red), Laminin (green) and Hoechst (blue). **c,d,** Body weights (**c**) and relative hindlimb muscle mass (**d**) to body weights of 16-month-old Notch^WT^ and Notch^ΔSC^ mice. **e,** Grip strength measurement of 16-month-old Notch^WT^ and Notch^ΔSC^ mice. **f,g** Running distance (**f**) and time (**g**) to exhaustion. (*p* = 0.3728 for **f,g**). Scale bars, 100 μm (**b**). Data presented are means ± s.e.m. Data shown are representatives of at least 3 independent experiments. n = 5 for Notch^WT^ and Notch^ΔSC^ mice (**c,d**), n = 4 and 6 for Notch^WT^ and Notch^ΔSC^ mice, respectively (**e**–**g**). 2-tailed Student’s *t-*test for **c,e,f,g** . 2-way ANOVA for **d**. *n.s.* non-significant.

**
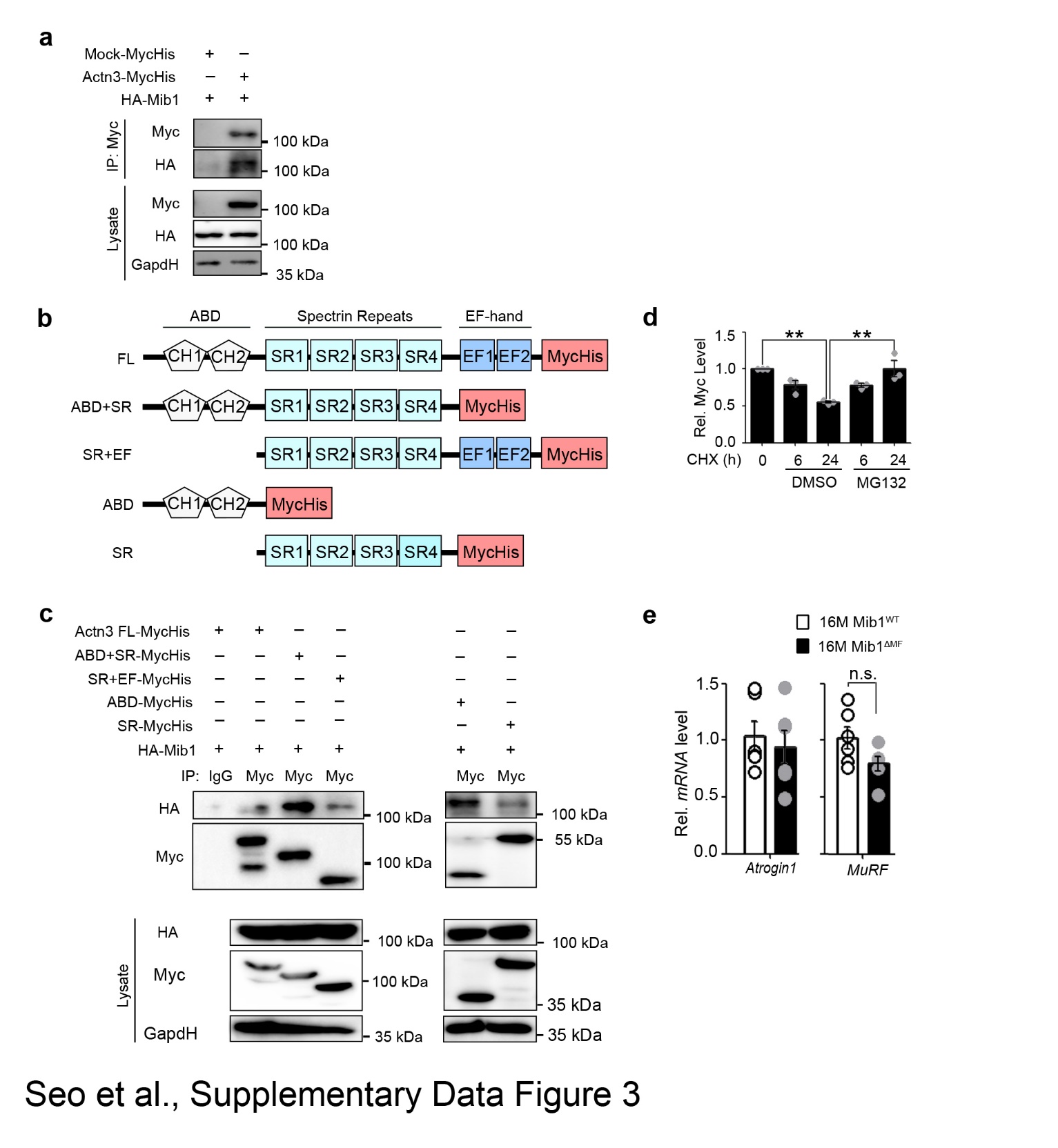
**

**Supplementary Fig 3. Interaction of Mib1 and Actn3 in skeletal muscles**

**a**, Co-immunoprecipitation (IP) of lysates from 293T cells overexpressing MycHis-tagged Actn3 and HA-tagged Mib1. Whole lysates were subjected to co-IP using anti-Myc, followed by IB analysis of Actn3 (Myc) and Mib1 (HA) levels. **b,** Schematic representation of domains structure of Actn3 and truncated constructs. FL, full length. ABD, actin-binding domain; SR, spectrin repeats; EF, EF-hand. **c,** IP of lysates from 293T cells overexpressing MycHis-tagged Actn3 FL and other Actn3 truncated constructs. Whole lysates were subjected to IP using Myc, followed by IB analysis of Actn3 (Myc) and Mib1 (HA) levels. **d,** The intensity of Actn3 protein degradation in 293T cells (corresponding to Fig.4d). 293T cells were transfected with MycHis-tagged Actn3 and HA-tagged Mib1 followed by treatment with cycloheximide (CHX) and MG132 or 0.1% DMSO for the indicated times. **e,** *Atrogin1/MAFbx,* and *MuRF* mRNA levels in GA muscles. (*p* = 0.08 for *MuRF*). Data presented are means ± s.e.m. Data shown are representatives of at least 3 independent experiments (**a**). n = 3 (**d**) and 6 (**e**) for each group. 1-way ANOVA for **d**. 2-tailed Student’s *t-*test for **e**. ***p*<0.01; *n.s*. non-significant.

**
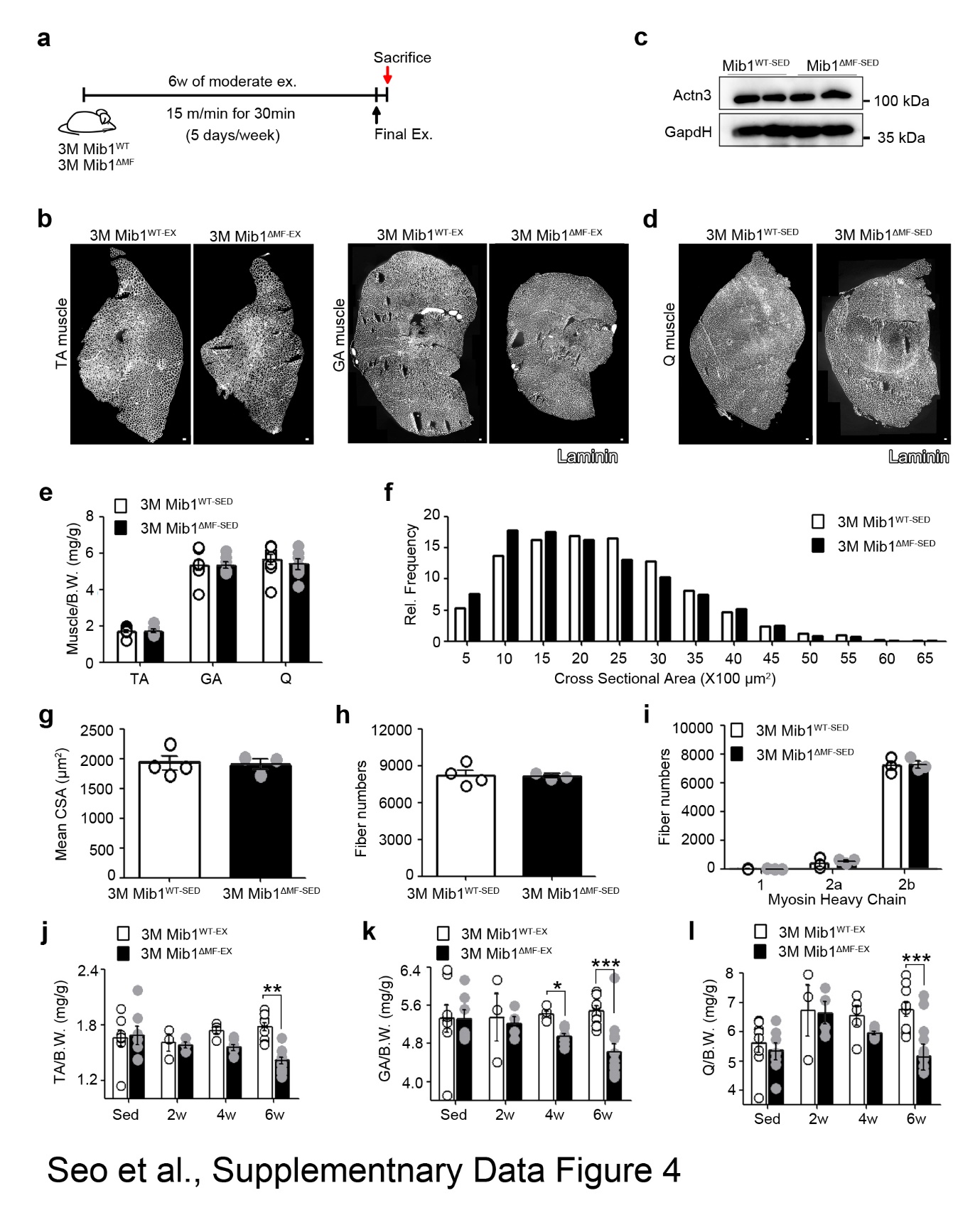
**

**Supplementary Fig 4. Normal skeletal muscles of young sedentary Mib1^ΔMF^ mice**

**a,** Three-month-old Mib1^WT^ and Mib1^ΔMF^ were subjected to chronic exercise with moderate intensity (15 m min^-1^. for 30 min.) for 5 days for 6 weeks and analyzed 24 hours after the final exercise. **b,** Cross-sections of TA (left panel) and GA (right panel) muscles of 3-month-old Mib1^WT-EX^ and Mib1^ΔMF-EX^ mice were subjected to IHC staining for Laminin (white). EX indicates exercised mice. **c,** IB analysis of Actn3 in GA muscles of sedentary 3-month-old Mib1^WT-EX^ and Mib1^ΔMF-EX^ mice. **d,** Cross-sections of Q muscles of sedentary 3-month-old Mib1^WT-SED^ and Mib1^ΔMF-SED^ mice were subjected to IHC staining for Laminin (white). SED indicates sedentary mice. **e,** Relative hindlimb muscle masses to body weights of 3-month-old Mib1^WT-SED^ and Mib1^ΔMF-SED^. **f,g,** Morphometric quantification of CSA (**f**) and mean CSA (**g**) of whole myofibers of Q muscle. **h,i,** Quantification of whole myofiber numbers (**h**) and myofiber numbers by fiber types (**i**) of Q muscle. **j**–**l** Relative TA (**j**), GA (**k**), and Q (**l**) muscles to body weights of 3-month-old Mib1^WT-EX^ and Mib1^ΔMF-EX^ mice at indicated time points of chronic exercise. 2w, 4w and 6w indicate 2-, 4-, 6-weeks of chronic exercise, respectively. Scale bars, 100 μm (**b,d**). Data presented are means ± s.e.m. Data shown are representatives of at least 3 independent experiments. n = 8 and 7 for Mib1^WT-SED^ and Mib1^ΔMF-SED^ mice, respectively (**e**). n = 4 and 3 for Mib1^WT-SED^ and Mib1^ΔMF-SED^ mice, respectively (**f**–**h**). n = 3 for Mib1^WT-SED^ and Mib1^ΔMF-SED^ mice (**i**). n = 8, 3, 5, and 10 for sed, 2w, 4w, 6w Mib1^WT-EX^ and 7, 4, 7, 12 for sed, 2w, 4w, 6w Mib1^ΔMF-SED^ mice, respectively (**j**–**l)**. Data shown are representatives of 3 independent experiments (**c**). 2-way ANOVA for **e**,**i**–**l**. *χ*^2^ test for trends for **f**. 2-tailed Student’s *t-*test for **g,h**. **p*<0.05; ***p*<0.01; ****p*<0.001.

**
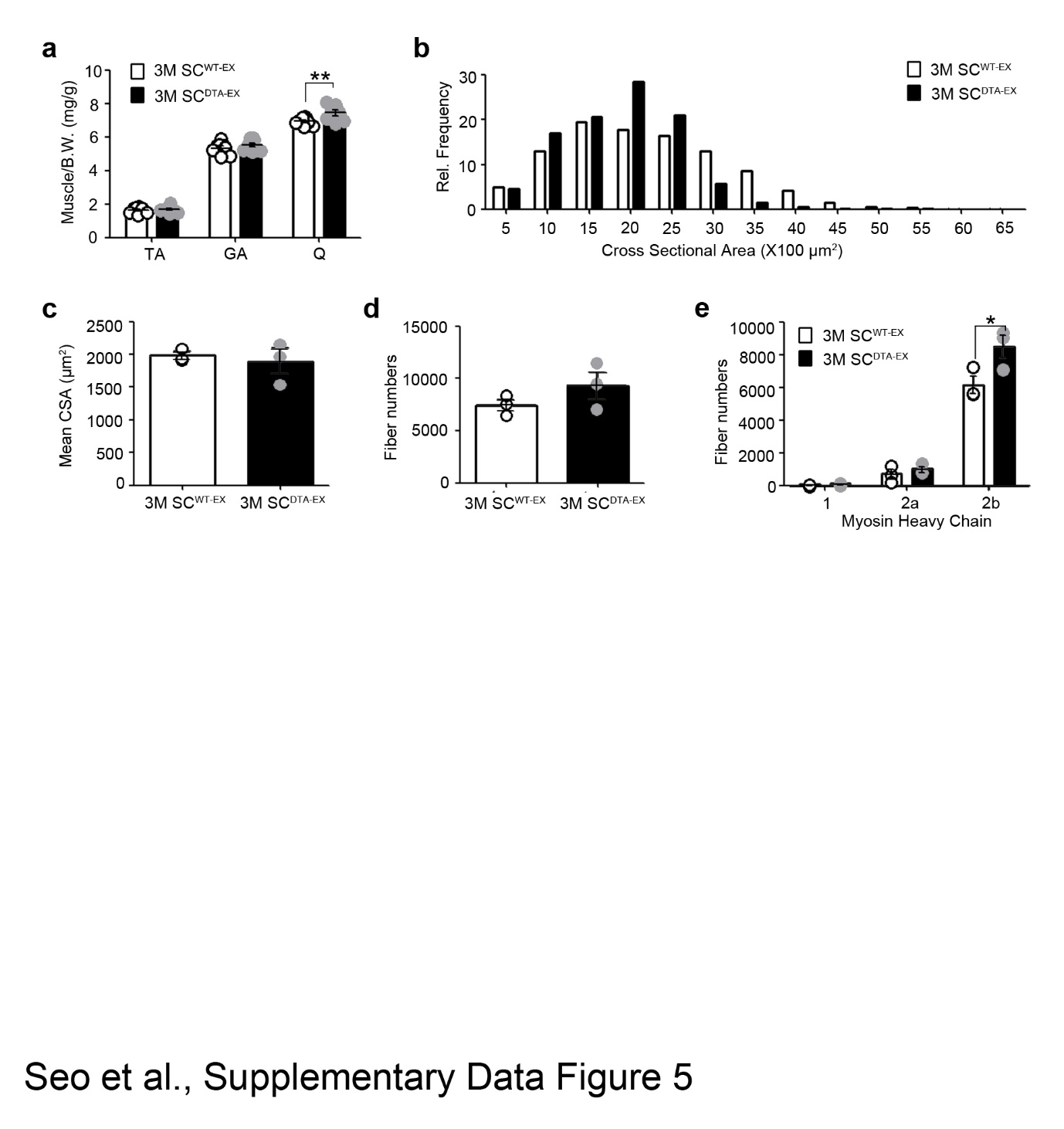
**

**Supplementary Fig 5. Normal skeletal muscles of chronically exercised young muscle stem cell-depleted mice**

Three-month-old mice were administered tamoxifen by daily oral for 5 consecutive days and analyzed at the age of 3 months after 6 weeks of chronic exercise. **a,** Relative hindlimb muscle masses to body weights of 3-month-old SC^WT-EX^ and SC^DTA-EX^ mice. EX indicates exercised mice. **b,c,** Morphometric quantification of CSA (**b**) and mean CSA (**c**) of whole myofibers. **d,e,** Quantification of whole myofiber numbers (**d**) and myofiber numbers by fiber types (**e**). Data presented are means ± s.e.m. Data shown are representatives of at least 3 independent experiments. n = 8 (**a**) or n = 3 (**b**–**e**) mice per genotype. 2-way ANOVA for **a,e**. *χ*^2^ test for trends for **b**. 2-tailed Student’s *t-*test for **c,d**. **p*<0.05; ***p*<0.01.


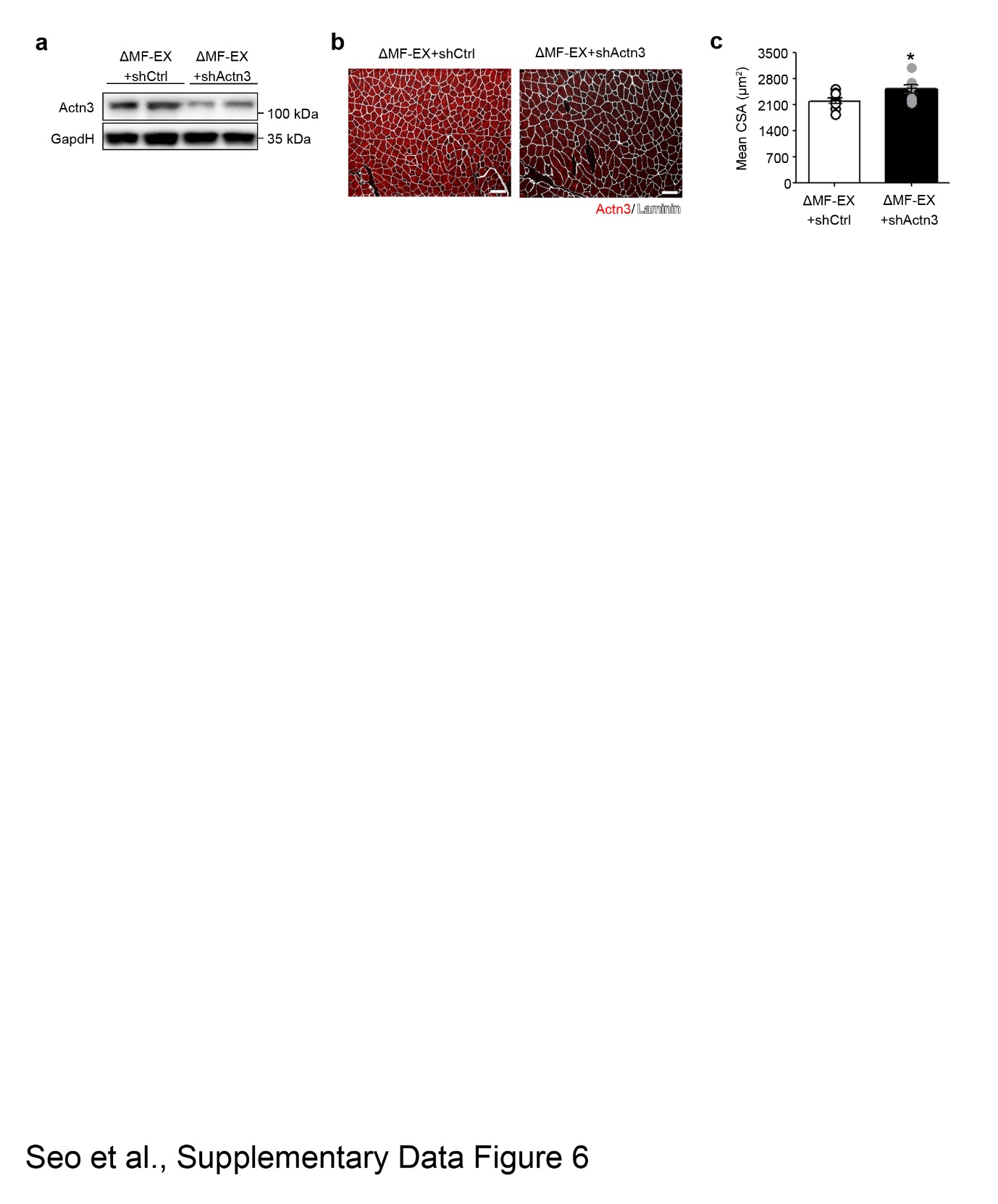


**Supplementary Fig. 6. Amelioration of chronic exercise-induced muscle atrophy of young Mib1^ΔMF^ mice**

**a,** IB analysis of Actn3 expression in Q muscles. **b,** Representative images of IHC staining for Actn3 (red) and Laminin (white). The IHC images were taken simultaneously with the same light setting, exposure time, and magnification. (Upper panels, low magnification; Low panels; high (inset) magnification). **c,** Mean CSA of type 2 glycolytic myofibers in Q muscles. (*p* = 0.0468). Scale bars, 125 μm. Data presented are means ± s.e.m. Data shown are representatives of at least 3 independent experiments. n = 4 (**a**) and 8 (**c**) per each genotype. 2-tailed Student’s *t*-test for **c**. **p*<0.05

**
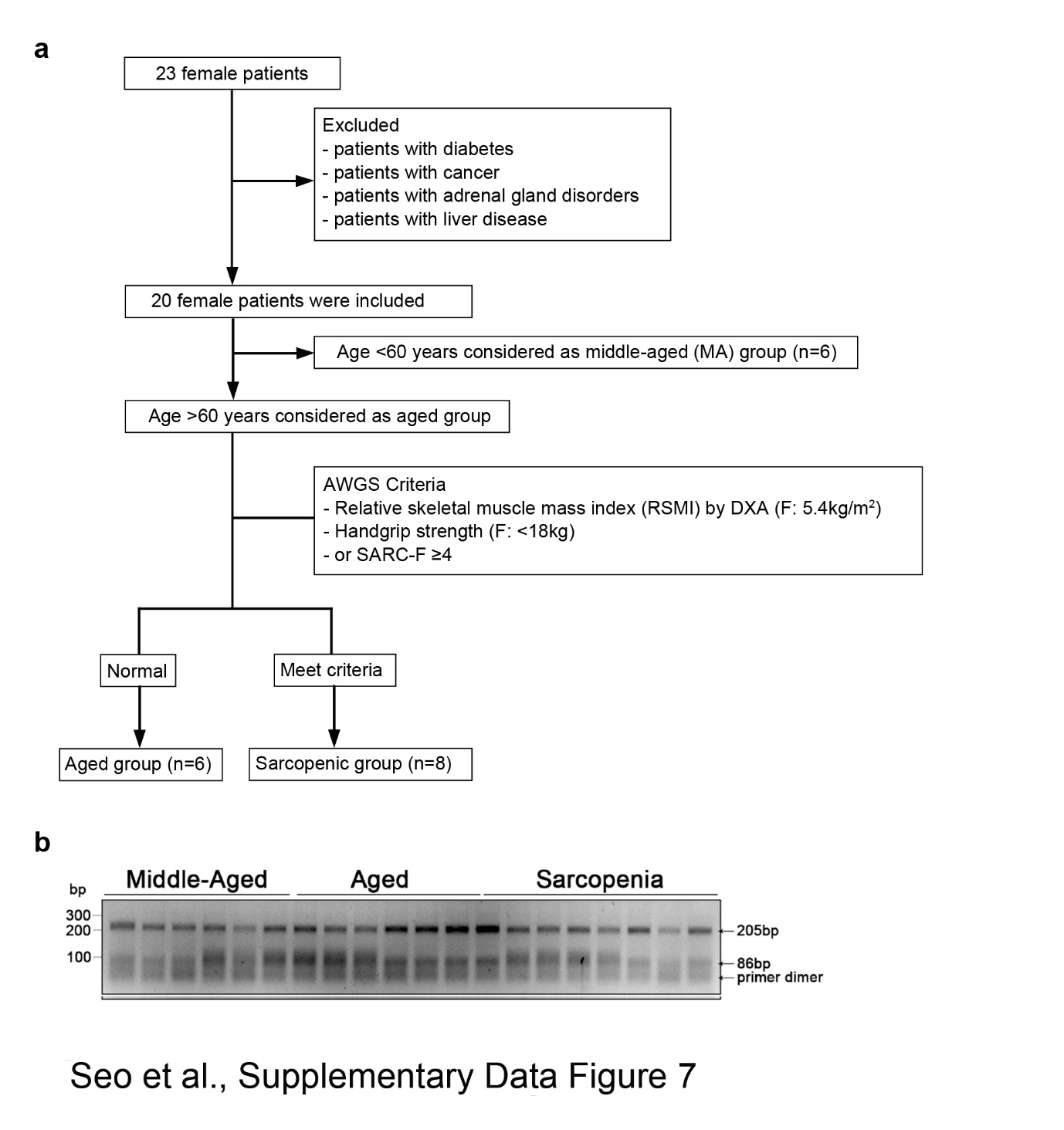
Supplementary Fig 7. Study cohort selection flow chart and Actn3 genotyping**

**a,** Flow chart for study cohort selection. **b,** Actn3 R577X genotyping. Note that RR type has fragments of 205 and 86bp while XX type has fragments of 108, 97, and 86bp. The arrows indicate 205bp, 86bp and primer dimer.

**Supplementary Table 1.** Mib1-binding protein candidates analyzed in the yeast two-hybrid assays using myofiber cDNA library

| **Gene symbol** | **Protein name** | **Identified time** | **Reporter Expression** | | | | **Type 2 (fast) myofiber specific expression** |
| --- | --- | --- | --- | --- | --- | --- | --- |
|  |  |  | **MEL** | **AUR-C** | **HIS3** | **ADE2** |  |
| Actn3 | Alpha-actinin3 | 3 | **+++** | **+++** | **+++** | **+++** | Yes |
| Eno3 | Beta-enolase | 1 | **++** | **+++** | **+++** | **+++** | Yes |
| Trim76 | Tripartite motif-containing protein 76 | 1 | **++** | **++** | **++** | **++** | No |
| Palladin | Palladin, cytoskeletal associated protein | 5 | **+++** | **+++** | **+++** | **+++** | No |
| Pebp1 | Phosphatidylethanolamine-binding protein | 3 | **+++** | **+++** | **+++** | **+++** | Yes |
| Snapap | SNARE-associated protein snapin | 1 | **+++** | **+++** | **+++** | **+++** | No |
| Ankrd26 | Ankyrin repeat domain-containing protein 26 | 1 | **+++** | **+++** | **+++** | **+++** | No |
| Cep72 | Centrosomal protein 72 | 3 | **+++** | **+++** | **+++** | **+++** | No |
| Ppp1r18 | Protein phosphatase 1 F-actin cytoskeleton-targeting subunit | 1 | **+++** | **+++** | **+++** | **++** | No |
| Atp6v0b | ATPase H^+^ transporting V_0_ subunit B | 1 | **+++** | **+++** | **+++** | **++** | No |
| Yars | Tyrosine-tRNA ligase, cytoplasmic | 1 | **+++** | **+++** | **+++** | **+++** | No |
| Lrrcc1 | Leucine rich repeat and coilied-coil domain containing 1 | 1 | **+++** | **+++** | **+++** | **+++** | No |
| Fam171b | Family with sequence similarity 171 member B | 1 | **+++** | **+++** | **+++** | **+++** | No |
| CDK5 | Cyclin-dependent kinase 5 | 1 | **+++** | **+++** | **+++** | **++** | No |
| Tim13 | Translocase of inner mitochondrial membrane 13 homolog |  | **+** | **++** | **++** | **++** | No |
| Hnrnpr | Heterogenous nuclear ribonucleoprotein R | 1 | **++** | **+++** | **+++** | **+++** | No |
| Elf2S2 | Eukaryotic translation initiation factor 2b, subunit 2 beta | 1 | **+++** | **+++** | **+++** | **+++** | No |
| Eed | Embryonic ectoderm development | 1 | **+++** | **+++** | **+++** | **+++** | No |
| Ubxn1 | UBX domain containing 1 | 1 | **+++** | **+++** | **+++** | **+++** | No |
| Usp1 | Ubiquitin specific peptidase 1 | 1 | **++** | **++** | **++** | **++** | No |
| Ube2d3 | Ubiquitin-conjugating enzyme E2D3 | 2 | **++** | **+** | **+** | **+** | No |
| Ccdc14 | Coiled coil domain containing 14 | 1 | **+++** | **+++** | **+++** | **+++** | No |
| Ccdc61 | Coiled-coil domain containing 61 | 1 | **+++** | **++** | **++** | **++** | No |

| **Characteristics** | **Middle-Aged** | **Aged** | **Sarcopenia** | ***P* value** |
| --- | --- | --- | --- | --- |
| Participants (n) | 6 | 6 | 8 |  |
| Age, yr | 46.00±3.28 | 75.67±3.9 | 83.75±2.01 | <0.0001 |
| Height, cm | 160.87±2.21 | 150.27±2.07 | 148.46±2.01 | <0.01 |
| Weight, kg | 59.12±2.66 | 50.68±1.22 | 46.29±3.36 | <0.05 |
| BMI, kg/m^2^ | 22.97±1.48 | 22.38±1.22 | 20.91±1.24 | 0.1774 |
| RSMI (kg/m^2^) |  | 5.67±0.23 | 5.30±0.29 | 0.0818 |
| Grip strength (kg) | 22.33±0.80 | 19.75±0.87 | 9.31±1.50 | <0.0001 |
| Sarc-F | 0.00 | 3.00±1.26 | 4.88±0.64 | <0.01 |

**Supplementary Table 2.** Characteristics of participants, body compositions and skeletal muscle index in female patients who underwent hip joint surgery

BMI, body mass index; RSMI: relative skeletal muscle index (lean mass of arms [kg]+lean mass of legs [kg])/ (height [m^2^]). 2-tailed Student’s *t*-test for RSMI. 1-way ANOVA for height, weight, BMI, grip strength, and Sarc-F. Data presented are means ± s.e.m.

**Supplementary Table 3.** The sequences of cDNA and shRNA primers

| Sequences for cDNA and shRNA primers | | Gene Bank Accession # |
| --- | --- | --- |
| Mib1-Forward | CCTACGACCTGCGTATCCTG | NM_001364997.1 |
| Mib1-Reverse | ACCTTTCCTCTACGCCCATT | NM_001364997.1 |
| Actn3-Forward | ATATCGTGAACACCCCCAAA | NM_013456 |
| Actn3-Reverse | TCCACTCCAACAGCTCACTG | NM_013456 |
| Gapdh-Forward | AACTTTGGCATTGTGGAAGG | NM_001289726 |
| Gapdh-Reverse | ACACATTGGGGGTAGGAACA | NM_001289726 |
| Atrogin1-Forward | GTCGCAGCCAAGAAGAGAAAGA | NM_026346.3 |
| Atrogin1-Reverse | TGCTATCAGCTCCAACAGCCTT | NM_026346.3 |
| MuRF1-Forward | TAACTGCATCTCCATGCTGGTG | NM_001039048.2 |
| MuRF1-Reverse | TGGCGTAGAGGGTGTCAAACTT | NM_001039048.2 |
| Actn3 shRNA #1 | TCTGTACGTTGACATTGCG |  |
| Actn3 shRNA #2 | TGTGGAAGCGCATCTTGCC |  |
| Actn3 shRNA #3 | TCACAGCTAAGACCTTGCA |  |
| Actn3 shRNA #4 | TCAATGTTCTCGATCTGCG |  |
| shRNA control | CCCGCCTGAAGTCTCTGATTAA |  |
