# Supplementary material for: Maintenance of type 2 glycolytic myofibers with age by Mib1-Actn3 axis
Source: Nat Commun. 2021 Feb 26;12:1294. doi: 10.1038/s41467-021-21621-6 (PMC7910585; doi:10.1038/s41467-021-21621-6)
Supplement: Supplementary file 2 — Reporting Summary [file 41467_2021_21621_MOESM2_ESM.pdf]

nature research

Corresponding author(s): Young-Yun Kong

Last updated by author(s): Jan 6, 2021

Reporting Summary

Nature Research wishes to improve the reproducibility of the work that we publish. This form provides structure for consistency and transparency in reporting. For further information on Nature Research policies, see our Editorial Policies and the Editorial Policy Checklist.

Statistics

For all statistical analyses, confirm that the following items are present in the figure legend, table legend, main text, or Methods section.

n/a	Confirmed	
☐	☒	The exact sample size (*n*) for each experimental group/condition, given as a discrete number and unit of measurement
☐	☒	A statement on whether measurements were taken from distinct samples or whether the same sample was measured repeatedly
☐	☒	The statistical test(s) used AND whether they are one- or two-sided<br>*Only common tests should be described solely by name; describe more complex techniques in the Methods section.*
☒	☐	A description of all covariates tested
☒	☐	A description of any assumptions or corrections, such as tests of normality and adjustment for multiple comparisons
☐	☒	A full description of the statistical parameters including central tendency (e.g. means) or other basic estimates (e.g. regression coefficient) AND variation (e.g. standard deviation) or associated estimates of uncertainty (e.g. confidence intervals)
☒	☐	For null hypothesis testing, the test statistic (e.g. *F*, *t*, *r*) with confidence intervals, effect sizes, degrees of freedom and *P* value noted<br>*Give P values as exact values whenever suitable.*
☒	☐	For Bayesian analysis, information on the choice of priors and Markov chain Monte Carlo settings
☒	☐	For hierarchical and complex designs, identification of the appropriate level for tests and full reporting of outcomes
☒	☐	Estimates of effect sizes (e.g. Cohen's *d*, Pearson's *r*), indicating how they were calculated

Our web collection on statistics for biologists contains articles on many of the points above.

Software and code

Policy information about availability of computer code

Data collection	Immunofluorescence were taken by Zeiss Observer Z1 fluorescent microscope and SPOT Flex camera. The images were acquired using SPOT imaging microscopy imaging software and EVOS FL auto cell imaging system. Muscle stem cells were isolated using FACS Aria III cell sorter. Rotor-Gene-Q pure-detection software was used to capture qRT-PCR data. Western blotting membranes were developed using the Fusion solo chemiluminescence imaging system. TEM images were captured using Tecnai G2 Spirity Twin transmission electron microscope.
Data analysis	Fiber cross-sectional area was calculated using Leopard software. Fiber cross sectional area and fluorescent intensity were analyzed and calculated using SMASH. Quantifications of western blots were performed using ImageJ (1.51k). Statistical analyses were performed using GraphPad Prim 5.01. The PCR products were analyzed using NCBI BLAST alignment search tool (http://blast.ncbi.nlm.nih.gov/Blast.cgi)

For manuscripts utilizing custom algorithms or software that are central to the research but not yet described in published literature, software must be made available to editors and reviewers. We strongly encourage code deposition in a community repository (e.g. GitHub). See the Nature Research guidelines for submitting code & software for further information.

Data

Policy information about availability of data

All manuscripts must include a data availability statement. This statement should provide the following information, where applicable:

- Accession codes, unique identifiers, or web links for publicly available datasets
- A list of figures that have associated raw data
- A description of any restrictions on data availability

The source data underlying Figsures and Supplementary Figures are provided as a Source Data file. Other data that supporting the findings of this study are available from the corresponding author upon reasonable request. Source data are provided with this paper.

Field-specific reporting

Please select the one below that is the best fit for your research. If you are not sure, read the appropriate sections before making your selection.

☒ Life sciences ☐ Behavioural & social sciences ☐ Ecological, evolutionary & environmental sciences

For a reference copy of the document with all sections, see nature.com/documents/nr-reporting-summary-flat.pdf

Life sciences study design

All studies must disclose on these points even when the disclosure is negative.

Sample size	No sample size calculation was performed. Sample sizes were chosen based on the previous work using similar methods (Kim et al. 2016, NCB; Yum et al. 2016. Cancer Res; Seong et al. 2018, Development), the experience of the authors with molecular and in vivo studies as published in many studies, and on the preliminary data from our laboratory. Sample sizes may vary depending on animal availability. For human study, experiments were designed to detect the change in the levels of proteins with age at 80% power (α=0.05). Sample sizes may vary depending on available size of resected tissues.
Data exclusions	For human data analysis, the participants with endocrine disorders have been excluded in order to avoid any negative effects of impaired endocrine on skeletal muscle (Endocrine disorder results in muscle atrophy) (Study cohort selection flow chart is shown in Extended Data Fig. 7a)
Replication	Experiments were repeated at least 3 times to ensure reproducibility.
Randomization	We collected measurements from all mutants and control animals with no exclusions and animals were allocated randomly into experimental groups. In case of human study, no randomization is applied because we only observed the endogenous expression levels of proteins in tissues within the groups.
Blinding	For cellular and mouse studies, the experiments were performed in a blinded fashion. For example, grip strength and endurance running tests were performed in a blinded fashion, individuals performing the tests were not unaware of mouse genotypes until the data analyses were completed.

Reporting for specific materials, systems and methods

We require information from authors about some types of materials, experimental systems and methods used in many studies. Here, indicate whether each material, system or method listed is relevant to your study. If you are not sure if a list item applies to your research, read the appropriate section before selecting a response.

Materials & experimental systems

n/a	Involved in the study	
☐	☒	Antibodies
☐	☒	Eukaryotic cell lines
☒	☐	Palaeontology and archaeology
☐	☒	Animals and other organisms
☐	☒	Human research participants
☒	☐	Clinical data
☒	☐	Dual use research of concern

Methods

n/a	Involved in the study	
☒	☐	ChIP-seq
☒	☐	Flow cytometry
☒	☐	MRI-based neuroimaging

Antibodies

Antibodies used

Rabbit anti-Actn3 (ab68204, EP2531Y, Lot GR97028-7), Abcam
Mouse anti-Mib1 (sc-393551, D-6, Lot F0217), Santa Cruz
Mouse anti-C-Myc (sc-40, 9E10, Lot F7219), Santa Cruz
Mouse anti-HA (sc-7392, F-7, Lot G1817), Santa Cruz
Normal Mouse IgG (sc-2025, Lot E3117), Santa Cruz
Normal Rabbit IgG (2729S, Lot 5), Cell signaling
Rabbit anti-GapdH (2118S, 14C10, Lot 10), Cell signaling
Rabbit anti-b-actin (A2066, Lot 097M4883V), Sigma-Aldrich
Mouse anti-FLAG (F1804, Lot SLBS3530V), Sigma-Aldrich
HRP-conjugated donkey anti-Mouse IgG (715-035-151, Lot AB_2340771), Jackson ImmunoResearch
HRP-conjugated donkey anti-Rabbit IgG (711-035-152, Lot AB_10015282), Jackson ImmunoResearch
HRP-conjugated anti-Mouse IgG (W4021, Lot 0000306114), Promega
HRP-conjugated anti-Rabbit IgG (W4011, Lot 0000306129), Promega
Mouse anti-Pax7, DSHB

	Mouse anti-MyHC type 1 (BA-A5), DSHB<br>Mouse anti-MyHC type 2a (SC-71), DSHB<br>Mouse anti-MyHC type 2b (BF-F3), DSHB<br>Mouse anti-MyHC type 2x (6H-1), DSHB<br>Mouse anti-MyHC type 2x (6H-1), DSHB<br>Rat anti-Laminin (ab11576, 4H82, Lot GR268487-16), Abcam<br>Alexa Fluor 488-conjugated anti-Mouse IgG (A11029, Lot 1664758), Invitrogen<br>Alexa Fluor 594-conjugated anti-Mouse IgG (A11032, Lot 1826426), Invitrogen<br>Alexa Fluor 488-conjugated anti-Mouse IgM (A10680, Lot 1917945), Invitrogen<br>Alexa Fluor 488-conjugated anti-Rat IgG (A11007, Lot 1903506), Invitrogen<br>Alexa Fluor 594-conjugated anti-Rat IgG (A11006, Lot 11774053, Invitrogen<br>Alexa Fluor 594-conjugated anti-Rabbit IgG (A11037, Lot 1694755), Invitrogen
Validation	Antibodies were characterized and validated for use by their respective manufacturers. All commercial antibodies were validated for the application (WB, IHC-P, IP, IF, ElLISA and/orFlow Cyto) and the species (mouse, rabbit, rat, human, equine, canine, bovine, porcine, avian, goat, and/or monkey).

Eukaryotic cell lines

Policy information about cell lines

Cell line source(s)	HEK293T cell was obtained from ATCC.
Authentication	None of the cell lines used were authenticated
Mycoplasma contamination	All of the cell lines have been tested that negative Mycoplasma contamination.
Commonly misidentified lines<br>(See ICLAC register)	There is no use the misidentified lines.

Animals and other organisms

Policy information about studies involving animals; ARRIVE guidelines recommended for reporting animal research

Laboratory animals	All mice were on C57BL6/J background. MCK-Cre;Mib1f/f and Pax7-CreER; Rosa-DTA/+ male mice were used in this study. MCK-Cre, Pax7-CreER, Rosa-DTA, Notch1f/f, Notch2f/f, and Rbpjkf/f mice were purchased from the Jackson Laboratory. Mib1 mice were previously generated by Koo et al. (B.K. Koo et al., Development. Mind bomb 1 is essential for generating functional Notch ligands to activate Notch. 132. 3459-3470. (2005)). Male mice were used in this study. 3, 16, 24, and 30-month-old wild type mice were used. 3, 9, and 16-month-old MCK-Mib1f/f and Mib1f/f mice were used. 3 and 16-month old Pax7-Rosa-DTA and Rosa-DTA mice were used. 16-month-old Pax7-Rbpjf/f, Rbpjf/f, Pax7-Notch1f/f;Notch2flf, and Notch1f/f;Notch2f/f mice were used. For chronic exercise study, 3-moth-old MCK-Mib1f/f, Mib1f/f, Pax7-RosaDTA, Rosa-DTA mice were subjected to chronic exercise and sacrificed after 6 weeks. All of the mice were housed in a 12-hour light/12-hour dark cycle at room temperature of ~22 celsius with 40-60% humidity, and handled according to the guidelines of ethical committees at Seoul National University.
Wild animals	The study did not involve wild animals.
Field-collected samples	No field-collected samples were used.
Ethics oversight	All animal studies received ethical approval from the Institutional Animal Care and Use Committee (IACUC) at Seoul National University, Korea.

Note that full information on the approval of the study protocol must also be provided in the manuscript.

Human research participants

Policy information about studies involving human research participants

Population characteristics	Characteristics of participants, body compositions and skeletal muscle index in female patients who underwent hip joint surgery in the study are summarized in Supplementary Table 2.
Recruitment	Incised and marginally resected Vastus Lateralis muscle (average 0.5±0.1g) tissue samples were obtained from female patients who underwent hip joint surgery performed by Dr. Yong Chan Ha from Chung-Ang University College of Medicine (Department of Orthpaedic Surgery). No pre-selection criteria were applied except for participants with endocrine disorders. Participants with endocrine disorders were excluded due to negative impacts on skeletal muscle (see Extended Data Fig.7a; Endocrine disorders can result in muscle atrophy).
Ethics oversight	Study was approved by the institutional review board of Chung-Ang University College of Medicine (IRB number: 1981-007-383) and performed in accordance with relevant guidelines and regulations. Subjects provided written informed consent prior to participations.

Note that full information on the approval of the study protocol must also be provided in the manuscript.
